# A Rare Case of Pyoderma Gangrenosum in a Patient With Pancreatic Neuroendocrine Tumor

**DOI:** 10.7759/cureus.12343

**Published:** 2020-12-28

**Authors:** Jacqueline T Wesolow

**Affiliations:** 1 Internal Medicine, Moffitt Cancer Center, Tampa, USA

**Keywords:** pyoderma gangrenosum

## Abstract

Pyoderma gangrenosum is a rare inflammatory skin disease usually associated with an underlying internal condition. Diseases most commonly linked with pyoderma gangrenosum are inflammatory bowel disease (ulcerative colitis and Crohn’s disease), rheumatoid arthritis, and hematologic malignancies such as leukemia and lymphoma. In rare instances, it can occur as a type of paraneoplastic syndrome in patients with cancer. The most common cancers associated with pyoderma gangrenosum are breast cancer followed by rectal, gastric, renal, and lung cancers. There are scarce reports of neuroendocrine tumors associated with pyoderma gangrenosum. We discuss a case of a pancreatic neuroendocrine cancer patient afflicted with pyoderma gangrenosum.

## Introduction

Pyoderma gangrenosum is a rare inflammatory skin condition marked by a rapidly developing skin ulcer. It most commonly occurs on the legs, but it can develop anywhere on the body. We present a case of a patient with pancreatic neuroendocrine cancer who presented with biopsy-confirmed pyoderma gangrenosum on her arm. What makes this case unique is the association of pyoderma gangrenosum with neuroendocrine tumor, although it is usually linked with other conditions such as inflammatory bowel disease (IBD) and blood disorders.

## Case presentation

A 55-year-old Caucasian female was diagnosed with pancreatic neuroendocrine tumor (VIPoma) in 2014. She has never smoked cigarettes and denied use of recreational drugs or alcohol. She began treatment with octreotide in February 2014. She had mild disease progression and underwent peptide receptor radionuclide therapy (PRRT). She received three cycles of PRRT. Her follow-up scans showed a mixed response with mild increase and decrease in certain areas; therefore, chemotherapy with capecitabine and temozolomide was started in 2016. She then underwent a Whipple procedure in 2019 followed by radiation therapy and is currently being treated with monthly lanreotide. She presented to our inpatient cancer center as a transfer for further management of a right arm wound. She initially presented to the outside hospital with a fever of 103°F (39.4°C) and a wound on her right forearm that progressed rapidly from a scratch two days prior. She was treated for presumed cellulitis with intravenous vancomycin. Infectious work-up was unremarkable with negative wound and blood cultures. The lesion was biopsied. The patient was evaluated by an infectious disease specialist who de-escalated antibiotics to doxycycline 100 mg twice daily for 10 days with a slow steroid taper while awaiting final biopsy results. The patient was transferred to our hospital for further evaluation and management of her persistent right arm wound.

**Figure 1:**
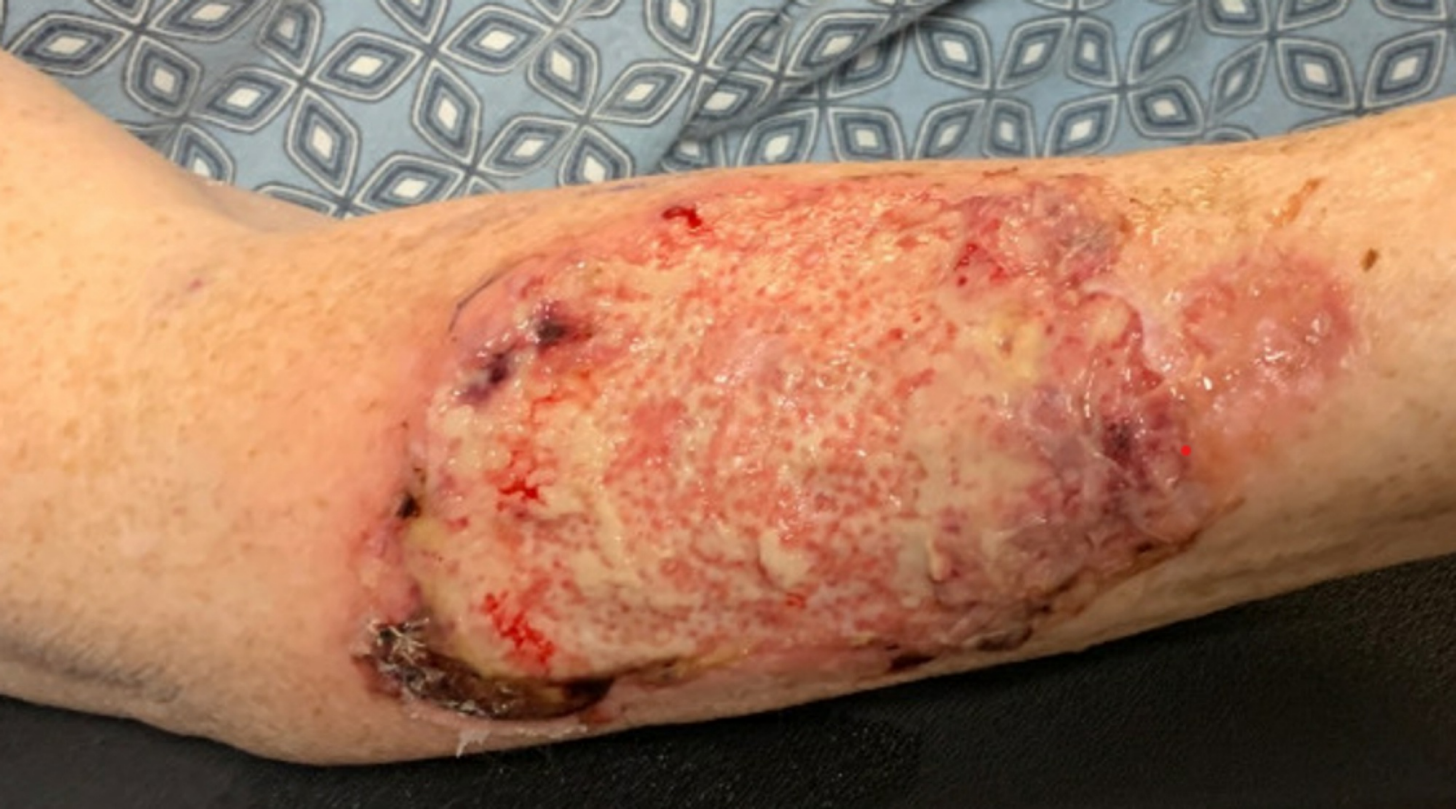
Pyoderma gangrenosum ulcerative lesion on the right forearm on initial admission to the hospital

**Figure 2:**
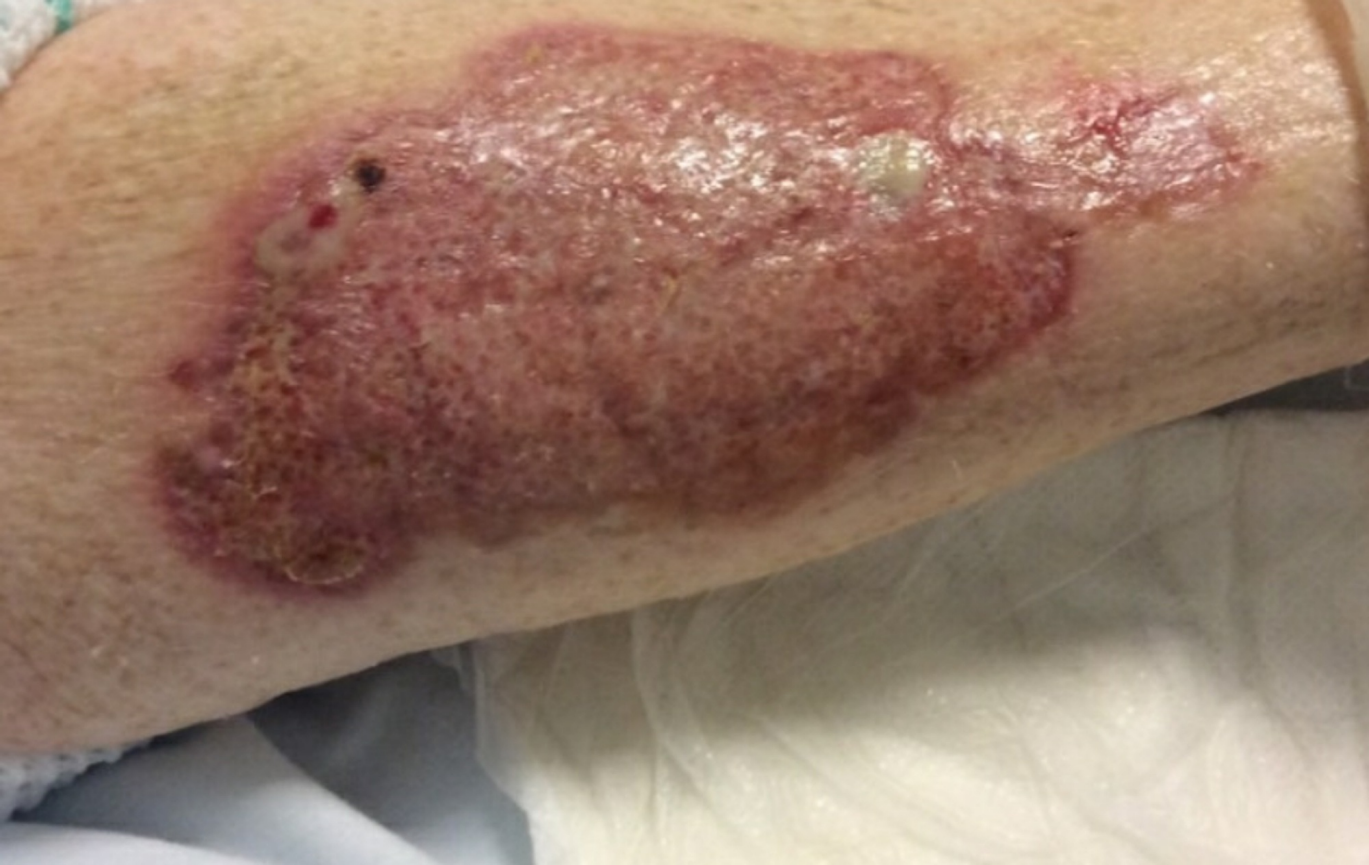
Post-inflammatory changes during the third week of steroid treatment

On admission to our facility, her vital signs were normal and she was afebrile. On laboratory work-up, her complete blood count showed the following: white blood cell count of 23.19 k/uL, hemoglobin of 7.7 g/dL, hematocrit of 23.6%, and platelet count of 55 k/uL. Blood cultures were negative, lactic acid was within normal limits, and wound culture showed no organisms. A respiratory viral panel was negative. Her infectious work-up was unremarkable. On physical examination, she was noted to have a large, painful ulcerated wound on the anterior aspect of her right forearm with elevated violaceous borders (Figure 1). The final biopsy report confirmed pyoderma gangrenosum. Dermatology recommended daily dressing changes with Vaselineä gauze followed by Telfaä, then Kerlixä, and then tape. The wound was kept clean with warm soap and normal saline. After approximately two to three weeks of steroid therapy, a vast improvement in the appearance of the ulcer was noted. The patient reported less pain. Her lesion significantly improved on prednisone and was marked by the appearance of post-inflammatory hyperpigmentary changes during her third week on steroids (Figure 2). Her lesions were healing well, and she was stable for discharge. She was prescribed 100 mg daily of oral prednisone to taper by 10 mg every five days for four weeks on discharge. The patient was scheduled for an outpatient clinic follow-up visit, but unfortunately she missed her follow-up appointment.

## Discussion

Pyoderma gangrenosum is a rare inflammatory skin disease first recognized in the early 1930s [1]. The estimated incidence is 3-10 patients per million population per year [2]. It mainly occurs in young to middle-aged adults (20-50 years old), with women more affected than men [2]. The pathogenesis remains poorly understood. It is marked by the presence of a painful ulcer that typically appears on the legs, although it can appear on any part of the body [1]. It is typically a diagnosis of exclusion due to its resemblance to other diseases such as herpes simplex virus type 2 and various types of vasculitis [3]. It is typically associated with an underlying condition such as IBD, rheumatoid arthritis, spondylitis, and some hematologic cancers. Some of the known hematologic cancers to be associated with pyoderma gangrenosum include chronic myeloid leukemia, acute myeloblastic leukemia, myelodysplasia, and lymphoma [2]. There are scarce reports of pyoderma gangrenosum occurring in neuroendocrine tumors. One such report describes pyoderma gangrenosum in a patient with carcinoid tumor of the ileum [4]. This particular patient was found on autopsy to have carcinoid tumor of the ileum [4].

A possible hypothesis for the link between carcinoid tumor and pyoderma gangrenosum is likely at the cellular level, although no investigations or studies have been conducted. In both conditions, we do see a disruption of immune cells. Immune cell infiltration, including B and T cells, mast cells, natural killer cells, and dendritic cells, has been reported in neuroendocrine tumors [5]. In pyoderma gangrenosum, infiltration of neutrophils is seen histologically. In both cases, a response at the immunological level has been described.

Our patient does not have known IBD, arthritis, or a hematologic malignancy. There are a few case reports in the literature on involvement of pyoderma gangrenosum in patients with solid organ tumors [4]. Another case discussed recurrence as a manifestation of a patient’s locally advanced breast cancer [6]. This patient did not have a clinical response to steroids; however, he had healing of her skin lesion following cancer treatment. Specially noted in this case was a direct correlation between the healing of the lesion and the patient’s cancer treatment. A literature review article published in 2019 found that breast cancer is the most commonly associated cancer linked to pyoderma, with a 31.6% association, followed by rectal (15.7%), gastric (10.5%), renal (10.5%), lung (10.5%), ileum (5.3%), hypopharyngeal (5.3%), colon (5.3%), and T-cell lymphoma (5.3%) [4].

Histologically, pyoderma gangrenosum is marked by an accumulation of neutrophils [7]. It is this neutrophil predominance along with studies that have found irregular neutrophil movement that are key characteristics of this condition [8]. Different diseases that present in a similar fashion are vasculitis (earlier known as Wegener’s granulomatosis), polyarteritis nodosa, Takayasu’s arteritis, primary cutaneous infections such as fungal infection, herpes simplex virus type 2, and exogenous tissue injury [8]. Data from a New England Journal of Medicine article state that the rate of misdiagnosis is 10%; therefore, it is recommended that a thorough work-up be performed along with a biopsy [9].

Treatment most commonly includes wound management, corticosteroids, and topical therapies [10]. Wound care includes keeping the ulcer clean and bandaged. Steroid therapy includes topical and oral forms [11]. Like in this patient case, it typically takes several weeks of therapy for lesions to heal depending on size. Smaller ulcers can be managed by topical steroids, tacrolimus ointment, dressings, and compression bandages. For larger ulcers, first-line treatment is oral steroids for 4 to 10 weeks with close monitoring [12].

## Conclusions

Pyoderma gangrenosum is a rare inflammatory skin disease marked by a rapidly progressive skin ulcer. Internal diseases most commonly linked are IBD such as Crohn's disease and ulcerative colitis, blood disorders such as leukemia and lymphoma, and autoimmune diseases. Therefore, in patients without known preexisting conditions who present with pyoderma gangrenosum, it is important to conduct a thorough physical and laboratory work-up to rule out underlying internal disease. Treatment is typically a combination of proper wound care, topical ointments, and steroids.
